# Ultrasound-assisted intralesional corticosteroid infiltrations for patients with hidradenitis suppurativa

**DOI:** 10.1038/s41598-020-70176-x

**Published:** 2020-08-07

**Authors:** Salvador-Rodriguez Luis, Arias-Santiago Salvador, Molina-Leyva Alejandro

**Affiliations:** 1 Dermatology Department, Hidradenitis Suppurativa Clinic, Hospital Universitario Virgen de Las Nieves, Avenida de Las Fuerzas Armadas 2, 18014 Granada, Spain; 2 European Hidradenitis Suppurativa Foundation (EHSF), Dessau-Roßlau, Germany; 3 Instituto de Investigación Sanitaria IBS Granada, Granada, Spain

**Keywords:** Health care, Medical research, Skin diseases

## Abstract

Corticosteroid infiltrations of lesions in hidradenitis suppurativa (HS) appear to be beneficial to acute flares. The aim of this study is to evaluate the effectiveness and safety of ultrasound-assisted intralesional corticosteroid infiltrations to HS lesions. Prospective cohort study between February 2017 and February 2019 on patients with mild to severe HS and one or more inflammatory lesions. The study intervention was ultrasound-assisted intralesional infiltration of triamcinolone acetonide 40 mg/ml. The main outcome was the complete response rate of infiltrated lesions versus non-infiltrated lesions. Two hundred and forty-seven infiltrated inflammatory lesions and 172 non-infiltrated lesions were included. At week 12, 81.1% (30/37) of nodules, 72.0% (108/150) of abscesses and 53.33% (32/60) of draining fistulas presented complete response versus 69.1% (47/68), 54.3% (38/70) and 35.3% (12/34) respectively for the non-infiltrated lesions. The Hurley stage negatively correlated with complete response for abscesses and draining fistulas at − 0.17 (SD 0.06) *p* < 0.01 and − 0.30 (SD 0.13) *p* < 0.02 respectively. Ultrasound-assisted corticosteroid infiltration is a useful technique for the treatment of inflammatory HS lesions, with high and sustained response rates, especially for abscesses and small to medium-size simple draining fistulas. The likelihood of response correlates negatively with the Hurley stage.

## Introduction

The administration of intralesional corticosteroids for the treatment of acute inflammatory lesions in patients with hidradenitis suppurativa (HS) is a commonly-used procedure in clinical practice. Current recommendations for their use are based on a multicentre study of 36 patients who only received intralesional corticosteroids to nodules or inflammatory abscesses with a follow-up period of 2 weeks^1^. They are also based on the latest review and recommendations of the HS Alliance working group who give the technique an evidence level 4 and a C grade recommendation, as well as indicating that its use is mainly for patients' symptomatic relief^2^.

In our experience, however, intralesional corticosteroids can achieve more than symptomatic relief if they are administered with the appropriate technique, and may produce long-term or permanent remission of treated lesions due to the fibrosis generated in dermis and subcutaneous tissue through the use of high-potency corticosteroids. The aim of this study is to evaluate the effectiveness and safety of ultrasound-assisted intralesional administration of corticosteroids to different types of inflammatory lesions in HS patients and to explore potential prognostic factors associated with a higher success rate.

## Patients and method

### Design and study population

Prospective cohort study carried out at the HS Clinic of the *Hospital Universitario Virgen de las Nieves* (HUVN) Granada, Spain, between February 2017 and February 2019.

The exposed cohort comprised the inflammatory lesions (inflammatory nodules, abscesses, draining fistulas) treated with intralesional corticosteroid infiltrations. The non-exposed cohort comprised the inflammatory lesions that remained untreated with intralesional corticosteroid infiltrations. Due to ethical reasons all patients with non-infiltrated lesions received concomitant systemic anti-inflammatory treatment.

### Inclusion criteria

(1) Patients older than 18 years with mild to severe HS, who were found to have one or more inflammatory lesions (inflammatory nodules, abscesses, draining fistulas) following physical and ultrasound examination and were candidates to be treated with intralesional corticosteroids. (2) Patients who had antibiotic or immunomodulatory treatment in the maintenance phase prior to the initial visit were included. (3) All patients gave informed consent for the technique to be carried out and to participate in the study.

The ethics committee of the HUVN approved this study in accordance with the Helsinki declaration.

### Exclusion criteria

(1) All patients who refused treatment with intralesional corticosteroids or did not want to participate in the study. (2) Patients who presented abscesses needing incision and drainage. (3) Patients who presented fistulas larger than 5 cm or more than one tract following ultrasound examination. (4) Patients who had started antibiotic treatment for HS (clindamycin +/− oral rifampicin, oral doxycycline, topical clindamycin), retinoids (acitretin) or corticosteroids within the 28-day period prior to the infiltration. (5) Patients who had started adalimumab treatment within the 12 week-period prior to the infiltration^3^. (6) Patients with signs of an active infectious process. (7) Patients who had undergone surgical procedures for HS to the treated area beyond incision and drainage in the previous 8 weeks. (8) Patients who presented allergy or hypersensitivity to the study medication.

### Study intervention

The study intervention consisted of intralesional administration of triamcinolone acetonide. Each lesion was injected guided by ultrasonography. Ultrasonography was performed with the use of a 7–15 MHz linear probe (myLab25 Esaote Spa, Genoa, Italy). The medication administered was triamcinolone acetonide at a dosage of 40 mg/ml. The maximum amount of triamcinolone acetonide administered per session was 40 mg. In each session, one or more lesions could be infiltrated. Each lesion was infiltrated only once. For patients who had multiple infiltration sessions, only lesions which had not been treated in previous sessions were candidates to be infiltrated. The choice of which lesions to treat was made according to clinical and ultrasound criteria^4^ and also patient discomfort, in order to achieve symptomatic relief from the lesions which most affected the patient's quality of life.

### Injection technique

**Figure 1:**
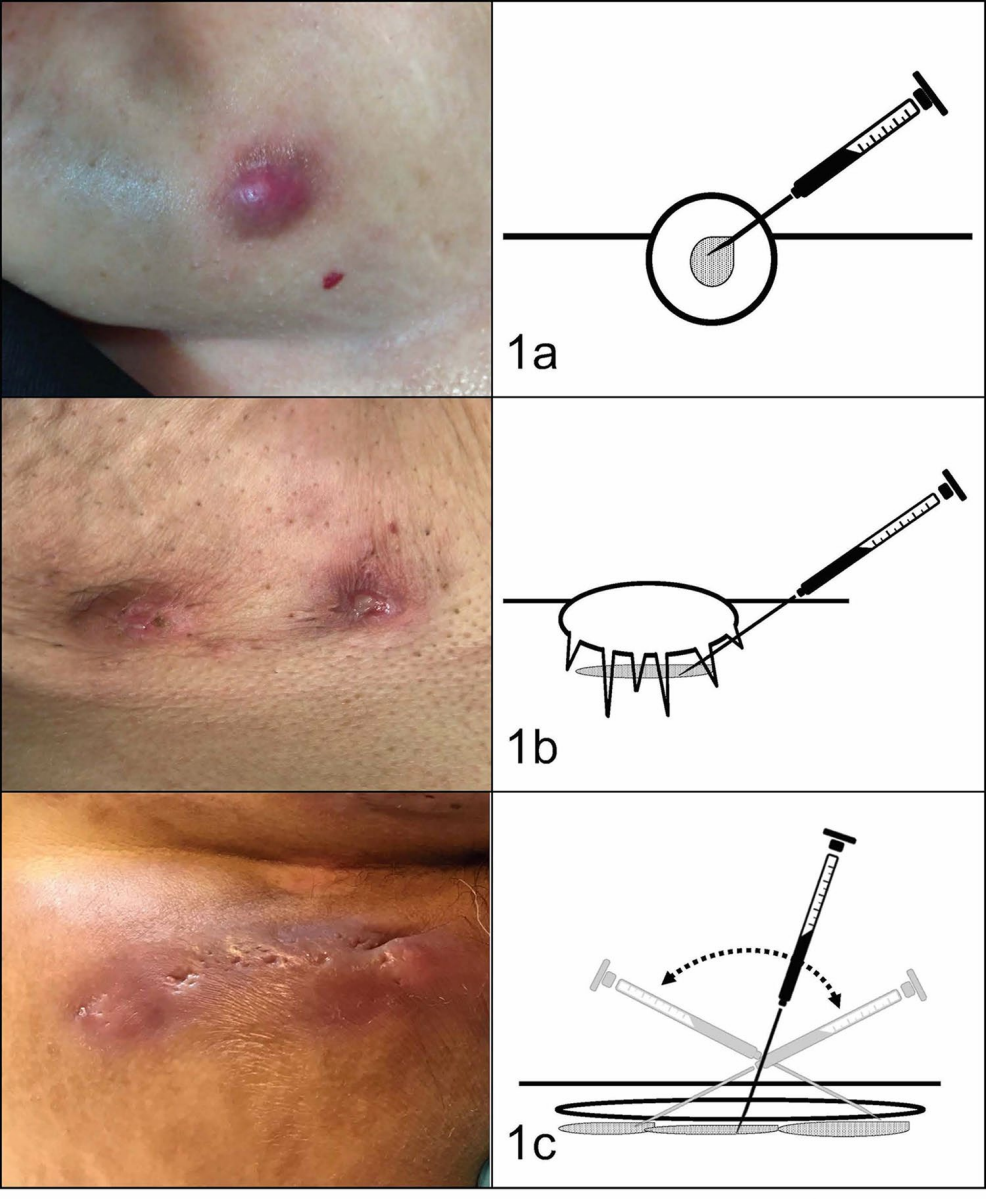
Corticosteroid infiltration technique. (**a**) Inflammatory nodules were injected directly inside the lesion. (**b**) Abscesses were injected beneath the base of the lesion in the area of panniculitis. (**c**) Fistulous tracts were injected with one or more injections along the base of the tract.

All the injections were performed by a dermatologist with experience in treating HS (AML). Infiltrations were performed with 1 ml syringes in combination with 21G needles. Figure 1 shows the exact area where the corticosteroids were injected.

### Variables of interest

The response was assessed 12 weeks after the administration of intralesional corticosteroids.

### Individual lesion response

The response was evaluated according to clinical and ultrasound criteria. Complete response in inflammatory nodules and abscesses was considered to be those cases where the lesion was completely resolved at follow-up: clinical examination showed no inflammation, pain or induration and ultrasound examination showed no Doppler signal or fluid collections. In draining fistulas, the absence of both suppuration and the Doppler signal at follow-up was considered as complete response.

### Overall patient response

Overall response was evaluated using objective and subjective measures. To evaluate the effect on global inflammatory load, the International Hidradenitis Suppurativa Severity Scoring System (IHS4) was used^5^. The formula to calculate IHS4 is: number of inflammatory nodules multiplied by 1+ number of abscesses multiplied by 2+ number of draining fistulae multiplied by 4. Results range from 0 to infinite. Scores under 4 indicate a mild inflammatory load; from 4 to 10 a moderate inflammatory load; and over 10 a severe inflammatory load. In the present study all scores refer to the counting of lesions identified by cutaneous ultrasonography^6^.

Subjective response was assessed using the Numeric Rating Scale for pain (NRS pain)^7^ and the patient's subjective perception of the severity of the disease (Patient Reported Severity, PRS). Both have a score that ranges from 0 and 10, where for NRS, 0 means absence of pain and 10 maximum pain, while for PRS, 0 corresponds to inactive disease and 10 to maximum disease activity.

### Safety

Adverse drug reactions were qualitatively recorded at the follow-up visit.

### Statistical analysis

Descriptive statistics were used to present the characteristics of the sample. The Kolmogorov–Smirnov test was used to check the normality of the variables. Continuous data was reported as the mean (standard deviation, SD) or median (25th–75th percentile), while nominal data was reported as a proportion (number)^8^. Group comparisons of non-normally distributed data were carried out using the Mann–Whitney U test, while normally distributed data was investigated using t-tests. The χ^2^ Test or Fisher’s Exact Test were used for qualitative variables when necessary. The t-test for matched pairs was used to evaluate IHS4, NRS and PRS changes. A multivariate logistic regression analysis was performed to explore predictors of response. Epidemiological and statistical criteria were used to model variable selection^9,10^. The effect of each exploratory variable on the model and its significance was studied. If the variable improved the model fit and adequacy (based on the likelihood ratio criteria and the significance of the parameter) it was included; otherwise, the variable was excluded. Different models were fitted with respect to the factors related to individual lesion response and patient global response. The model was checked for pair-wise interaction between covariates. Potential confounding covariates were studied using a change in significance of the parameters in the model or a change of 30% of their value. Significance was set at two tails, *p* < 0.05^8,11^. Statistical Analyses were performed using JMP version 14.1.0 (SAS institute, North Carolina, USA).

### Statement of ethics

The study was approved by the Hospital Universitario Virgen de las Nieves Ethics Committee and is in accordance with the World Health Organization Declaration of Helsinki. Patients gave their informed consent to participate in the study and for the disclosure of the results.

## Results

### Cohorts

The exposed cohort comprised 247 infiltrated lesions (37 inflammatory nodules, 150 abscesses and 60 draining fistulas) treated in 130 infiltration sessions, corresponding to 77 patients.

The non-exposed cohort comprised 172 non-infiltrated lesions (68 inflammatory nodules, 70 abscesses and 34 draining fistulas) treated in 63 sessions, corresponding to 39 patients.

**Figure 2:**
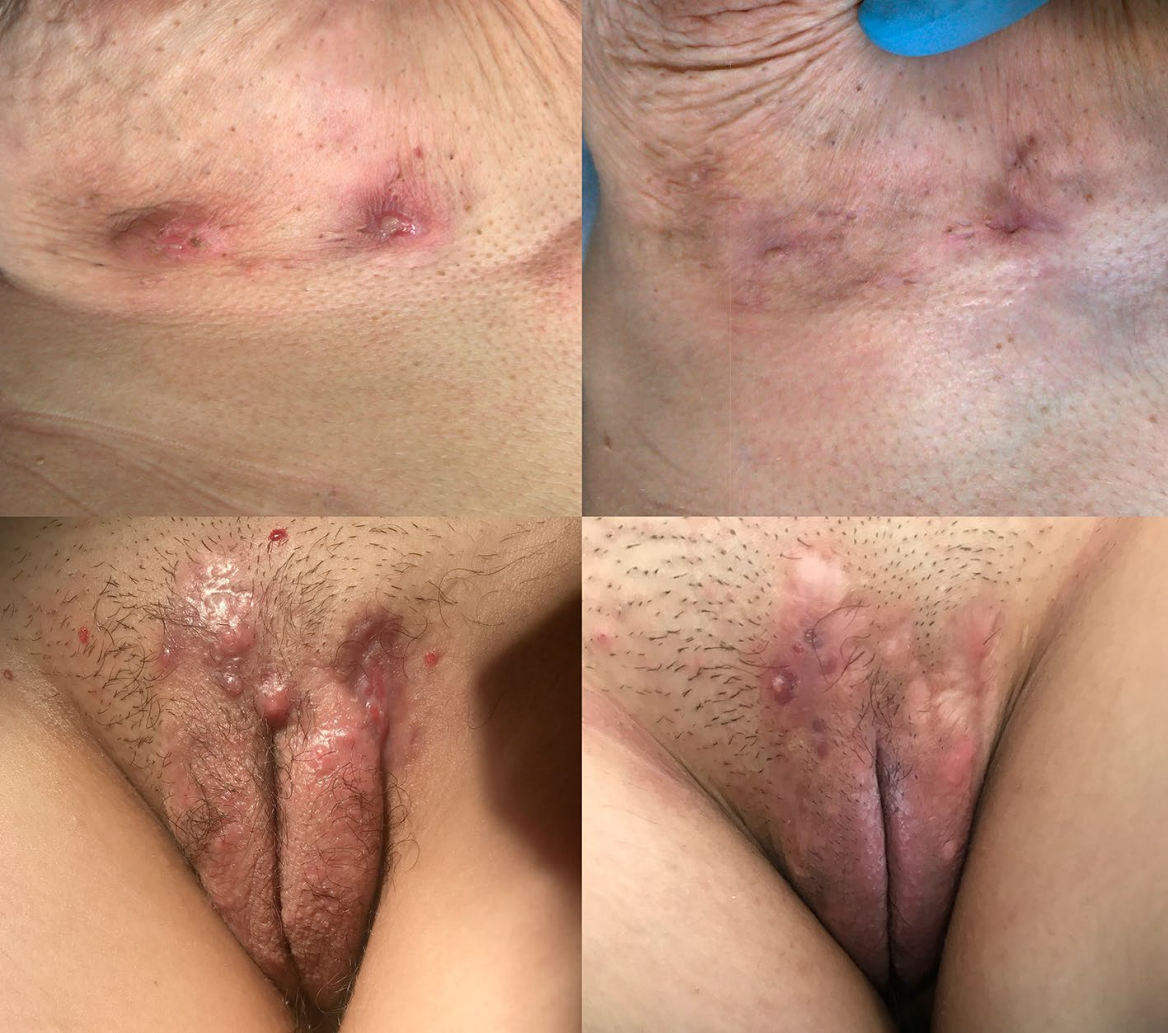
Clinical images of patients achieving complete clinical response at week 12 of lesions treated with ultrasound-assisted intralesional corticosteroids. Upper images: Abscesses in the submammary region infiltrated in monotherapy. Lower images: Abscesses and inflammatory nodules in the genital area infiltrated in the presence of concomitant systemic treatment.

The main characteristics of the sample are summarized in Table 1. The response rates at week 12 on infiltrated versus non-infiltrated lesions were 81.1% (30/37) versus 69.1% (47/68) *p* = 0.18 for inflammatory nodules, 72% (108/150) versus 54.3% (38/70) *p* = 0.009 for abscesses, and 53.4% (32/60) versus 35.3% (12/34) *p* = 0.09 for draining fistulas. Clinical images of infiltrated lesions which achieved complete response in monotherapy and in combination with systemic treatment are shown in Fig. 2.

**Table 1:** Demographic and disease-related characteristics of cohorts.

	Infiltrated cohort N = 130	Non-infiltrated cohort N = 63	*p*
Age	31.1 (SD 1.6)	35.5 (SD 1.1)	**0.02**
Sex (female:male)	45:18	89:41	0.67
Hurley I	26.9% (35)	23.8% (15)	0.4
II	54.6% (71)	49.2% (31)	
III	18.4% (24)	26.9% (17)	
Baseline IHS4	5.7 (SD 0.4)	7.4 (SD 0.6)	**0.01**
Systemic treatment	56.9% (74)	100% (63)	**0.01**

*IHS4* International Hidradenitis Suppurativa Severity Scoring System, *SD* standard deviation.

### General characteristics of the infiltrations

The majority of patients, 54.5% (42/77), only had one infiltration session, 28.6% (22/77) of patients had two infiltration sessions, 11.7% (9/77) of patients had three sessions, 3.9% (3/77) of patients four sessions and 1.3% (1/77) five sessions.

Regarding the features of the infiltrated lesions, the mean size of inflammatory nodules was 1.22 cm (SD 0.19), the mean size of abscesses was 2.43 cm (SD 0.32) and the mean size of the draining fistulas was 3.59 cm (SD 0.46). The most frequently infiltrated location was the axilla at 43.8% (57/130) followed by the groin at 41.5% (54/130), while the abdomen was the least frequent at 6.9% (9/130). The mean number of infiltrated lesions per session was 0.3 (0.9) inflammatory nodules; 1.2 (1.0) abscesses and 0.5 (0.7) fistulas. The mean number of infiltrated nodules and abscesses per session was similar in different Hurley stages *p* = 0.57 and *p* = 0.82 respectively; while the mean number of infiltrated fistulas per session was higher in patients with Hurley III versus II at 0.91 (0.14) versus 0.53 (0.08) *p* = 0.03.

Regarding concomitant treatment, 43.1% (56/130) of infiltrations were performed without associated anti-inflammatory treatment; 35.4% (46/130) were performed in the presence of systemic antibiotics; 8.4% (11/130) with systemic retinoids (acitretin); 7.7% (10/130) in the presence of biological treatment and 5.4% (7/130) in combination with two anti-inflammatory treatments. The baseline characteristics of the groups depending on whether the infiltrations were performed in monotherapy or combination are shown in Table 2.

**Table 2:** Characteristics of patients based on the modality of infiltration.

	Monotherapy (N = 56)	Combination (N = 74)	*p*
Age	36.7 (SD 1.7)	34.6 (SD 1.5)	0.37
Sex (female:male)	1:3	27:47	0.16
Hurley I	33.93% (19)	21.62% (16)	**0.0006**
II	62.5% (35)	48.65% (36)	
III	3.57% (2)	29.73% (22)	
Baseline IHS4	4.6 (SD 0.5)	6.6 (SD 0.4)	**0.009**
Baseline NRS pain	4.9 (SD 0.4)	4.9 (SD 0.3)	0.9
Baseline PRS	4.9 (SD 0.3)	5.09 (SD 0.3)	0.8
Total infiltrated nodules	0.46 (SD 0.11)	0.14 (SD 0.1)	**0.04**
Total infiltrated abscesses	1.14 (SD 0.1)	1.16 (SD 0.1)	0.9
Total infiltrated fistulas	0.3 (SD 0.09)	0.58 (SD 0.07)	**0.02**

Combination, infiltrations in combination with systemic treatments; IHS4, International Hidradenitis Suppurativa Severity Scoring System; Monotherapy, infiltrations in monotherapy; NRS, Numeric rating scale; PRS, patient reported severity; SD, standard deviation.

The univariate analysis of the factors associated with complete response according to the type of lesion is shown in Table 3. Regarding concomitant treatment, a sub-analysis was performed for each type of anti-inflammatory treatment (data not shown), and no significant differences were found between the different treatments studied.

**Table 3:** Univariate analysis of factors associated with individual lesion response.

	Likelihood of response in I.Nodules ß (SD)	*p*	Likelihood of response in Abscesses ß (SD)	*p*	Likelihood of response in D. Fistulas ß (SD)	*p*
Lesion size	1.23 (1.27)	0.34	− 0.16 (0.51)	0.75	− 0.004 (0.28)	0.88
Age	0.003 (0.006)	0.58	0.003 (0.003)	0.35	− 0.004 (0.006)	0.46
Sex Female–Male	− 0.12 (0.19)	0.53	− 0.02 (0.04)	0.64	− 0.14 (0.06)	**0.03**
Hurley stage	0.08 (0.13)	0.55	− 0.21 (0.06)	**0**.**007**	− 0.29 (0.13)	**0.04**
Baseline IHS4	0.016 (0.02)	0.56	− 0.03 (0.01)	**0**.**001**	− 0.02 (0.01)	**0**.**04**
Monot-Comb	0.15 (0.08)	0.10	0.06 (0.21)	0.76	− 0.005 (0.07)	0.94

ß, Beta; Comb, infiltrations in combination with systemic treatments; IHS4, International Hidradenitis Suppurativa Severity Scoring System; Monot, infiltrations in monotherapy; SD, standard deviation.

The multivariate logistic regression analysis R^2^ = 0.18 showed that the probability of complete response for abscesses positively correlated with the presence of concomitant systemic treatment, beta 0.08 (SD 0.04) *p* = 0.04 and negatively correlated with Hurley stage, beta − 0.17 (SD 0.06) *p* = 0.01 and baseline inflammatory load IHS4, beta − 0.02 (SD 0.01) *p* = 0.06.

In the case of draining fistulas, multivariate analysis R^2^ = 0.18 showed a higher probability of complete response in Hurley stage II versus III, beta − 0.30 (SD 0.13) *p* = 0.02 and in male sex, beta 0.14 (SD 0.06) *p* = 0.02. Variables included in the model were Hurley stage II–III, sex and age.

### Overall patient response

The overall reduction in IHS4 at follow-up was 2.2 (SD 3.6) *p* < 0.001; pain decreased by 1.5 (4.1) on the NRS scale, while PRS also decreased by 1.5 (3.4). The univariate analysis of factors potentially associated with a greater reduction in IHS4 is shown in Table 4.

**Table 4:** Univariate analysis of factors associated with overall objective and subjective improvement.

	IHS4 reduction ß (SD)	*p*	PAIN reduction ß (SD)	*p*	PRS reduction ß (SD)	*p*
Age	0.02 (0.02)	0.36	0.03 (0.02)	0.2	0.03 (0.02)	0.13
Sex Female–Male	0.49 (0.33)	0.14	0.23 (0.38)	0.53	0.35 (0.32)	0.26
Hurley stage	0.68 (0.46)	0.15	− 1.23 (0.52)	**0**.**02**	− 1.07 (0.44)	**0**.**01**
Baseline IHS4	0.42 (0.06)	**< 0**.**0001**	− 0.02 (0.08)	0.7	− 0.06 (0.06)	0.38
Monot.- Comb	0.14 0.31)	0.65	0.04 (0.36)	0.91	− 0.13 (0.30)	0.66

ß, Beta; Comb, infiltrations in combination with systemic treatment; IHS4, International Hidradenitis Suppurativa Severity Scoring System; Monot, infiltrations in monotherapy; PRS, patient reported severity; SD, standard deviation.

The multivariate analysis R^2^ = 0.29 showed that Hurley stage, beta − 1.22 (SD 0.47) *p* = 0.01 and a higher baseline IHS4, beta 0.53 (SD 0.07) *p* < 0.001 correlated with a greater reduction in IHS4. However, the probability of obtaining IHS4 score = 0 at follow-up, R^2^ = 0.43 was associated with a lower baseline IHS4, beta 0.46 (SD 0.07) *p* < 0.001 and lower Hurley stage, beta 1.16 (SD 0.48) *p* = 0.01, independent of the treatment modality monotherapy versus combined, beta 0.16 (SD 0.27) *p* = 0.55.

Multivariate analysis for pain improvement, R^2^ = 0.06 showed that Hurley stage, beta − 1.38 (0.52) *p* < 0.009 correlated with greater pain reduction. Multivariate analysis for Patient Reported Severity, R^2^ = 0.07 showed that Hurley stage, beta − 1.23 (0.44) *p* = 0.006 and age, beta 0.04 (0.02) *p* = 0.04 also correlated with a greater perceived improvement by the patient.

### Adverse reactions

Two systemic adverse reactions related to the medication were observed. A patient with type I diabetes suffered a glycaemic decompensation that was difficult to control for 3 weeks. Another patient experienced changes in behaviour, with a tendency towards aggressiveness, which was resolved in ten days.

## Discussion

The results of our study show that ultrasound-guided intralesional corticosteroid injections can improve inflammatory lesions in HS patients in the long-term, not only for inflammatory nodules and abscesses but also draining fistulas. However, in order to achieve this effect, a high-potency corticoid such as triamcinolone acetonide should be chosen and the infiltration should be placed appropriately. For this, the use of ultrasonography is crucial^12–14^.

Our study has several strengths. Firstly, the number of lesions studied is high. Secondly, we have evaluated the technique in different types of inflammatory lesions in patients with HS, analysing their individual response, including draining fistulas where scientific evidence is scarce. In addition, we have a medium-term follow-up period, which allowed us to analyse maintained responses and not only the short-term effect generated by the anti-inflammatory action of the corticosteroids after infiltration.

The most frequently infiltrated type of lesion was abscesses. This is probably due to the fact that this type of lesion produces a higher level of discomfort in the patient, which was one of the criteria when choosing which lesions to infiltrate.

There are several different predictors of individual lesion response. The type of lesion with the highest complete response rate was nodules, followed by abscesses and draining fistulas. This is probably due to the nature of the lesion, as nodules are lesions with a greater inflammatory component and less structural damage, which tend to resolve spontaneously. Consequently, no differences were observed between the response rate for infiltrated and non-infiltrated inflammatory nodules.

In the case of abscesses, the predictors of response were a lower Hurley stage, probably because abscesses which appear in patients with more advanced Hurley stages are recurrent and appear in anatomical areas where previous scar structures have been created, making it difficult to achieve complete resolution; lower baseline IHS4, since it implies that systemic inflammation is lower; and the presence of concomitant treatment, probably due to the synergic effect created between corticoid infiltrations and the anti-inflammatory effect of systemic drugs.

In the case of draining fistulas, it was also found that lower Hurley stage (II vs. III) was a predictor of response, probably for the same reason as the abscesses and also because the fistulas which appear in earlier Hurley stages are probably dermal fistulas which may have a better therapeutic response rate^15,16^. Male patients also showed a better likelihood of lesion response. It is important to note that fistulas larger than 5 cm or with multiple tracks were excluded and that the results refer to simple tract and small to medium-size draining fistulas.

A study has recently been published evaluating the use of intralesional triamcinolone acetonide in fistulas^17^. In this study the authors injected a mean of 0.49 ml triamcinolone 40 mg/ml in 46 patients, without a control group and with a subsequent follow-up of 90 days. The clinical and ultrasound complete response rate is similar to our study.

On the other hand, the predictors of a greater reduction in IHS4 are higher baseline IHS4 and Hurley stage, since the lesions treated in patients in this scenario are abscesses and draining fistulas. However, the probability of achieving complete disease control (IHS4 = 0) is greater in lower baseline IHS4 and at lower Hurley stages. Therefore, we can expect different outcomes when we perform intralesional corticosteroid infiltrations, depending on the patient’s baseline characteristics and the stage of the disease: either directed lesion improvement or complete disease control.

Regarding the subjective measures, we found that the improvement experienced in both pain and patient-reported severity is greater in patients with lower Hurley stages. Patients with low Hurley stages present less lesions and less structural damage so the main symptomatology is produced by acute inflammatory lesions. Treating these lesions can greatly alleviate patients’ symptoms.

The clinical trial NCT02781818 has recently been completed and its results have already been published. Researchers evaluated the effect of triamcinolone acetonide 10 mg/ml, 40 mg/ml or placebo in 58 lesions (only nodules and abscesses) for 2 weeks in 48 patients. They did not find any difference in the number of days for lesion resolution, or any change in pain or patient-reported severity between the different tails of the study. We consider that this low effectiveness is due to an insufficient (0.1 ml) dose of corticosteroid and because the infiltrations were not assisted by ultrasonography^18^.

On the other hand, a multicentre retrospective clinical study has recently been published evaluating the use of corticoid infiltrations in 135 lesions, including non-inflammatory nodules, inflammatory nodules, abscesses and fistulas. In this study, the authors found a high rate of successful response after the infiltrations, similar to that of our study. Skin ultrasound was performed prior to treatment on most lesions and the authors concluded that this ultrasound examination may improve clinical response in all types of lesions as it allows a more accurate measurement of the depth and location of skin lesions^19^. However, ultrasound-assisted infiltrations were performed in less than half of the treated lesions. Ultrasound was used in complex or larger lesions, which limits the generalizability of the results, since more complex lesions may have lower success rates due to the nature of the lesion rather than the procedure. In consequence, lower response rates were observed compared to non-ultrasound-assisted infiltrations.

Regarding safety, although intralesional injections are an easy procedure, care must be taken when performing them on diabetic patients, due to the possibility of producing glycaemic decompensation^20^. It would also be necessary to carry out an individual evaluation of the patient's mental health, given the potential of systemic corticosteroids to produce changes in behaviour, as already described in previous studies^21,22^.

Our study has some methodological limitations that should be taken into account: (1) The study was carried out using cutaneous ultrasonography to determine the type of lesion and to guide the procedure, which limits the generalization of the results to settings where ultrasound is not available. (2) Due to the observational design, the non-exposed cohort presents a higher baseline IHS4 value and a higher frequency of concomitant systemic treatment. Patients with a more severe condition are less likely to benefit from lesion-directed interventions like corticosteroid infiltrations and will need systemic treatment. However, these differences did not work in favour of the exposed cohort, in order to have more statistically significant differences. (3) The comparator group is composed of non-infiltrated lesions, which limits our ability to determine the added-value of ultrasound guidance for intralesional corticosteroids infiltrations. Future studies comparing both techniques are guaranteed.

In conclusion, we present the largest clinical study to date evaluating the effectiveness and safety of intralesional corticosteroid infiltrations in inflammatory lesions for HS patients in a real clinical practice setting and considering the type of lesion treated. The results of our study show that intralesional corticosteroid infiltrations can have a continued beneficial effect on abscesses and simple small to medium draining fistulas in HS patients. Evidence level 2b. We have observed a good response of treated lesions both in the presence and absence of anti-inflammatory treatment, improving both objective and subjective indexes of severity with a low rate of adverse effects. The Hurley stage of the patient inversely correlates with the probability of achieving complete resolution of the treated lesions, but the most severe patients are those who obtain the greatest reduction in inflammatory load. Therefore, the use of intralesional corticosteroids has a place in different profiles of patients with HS, always considering the real objectives which can be achieved with each patient depending on the baseline characteristics of their disease.

## Supplementary information

Supplementary information

## Data Availability

All data and material are available for readers in methods and supplementary information.
