# Publisher Correction: Ultrasound-assisted intralesional corticosteroid infiltrations for patients with hidradenitis suppurativa

**DOI:** 10.1038/s41598-020-74153-2

**Published:** 2020-10-13

**Authors:** Luis Salvador‑Rodriguez, Salvador Arias‑Santiago, Alejandro Molina‑Leyva

**Affiliations:** 1 Dermatology Department, Hidradenitis Suppurativa Clinic, Hospital Universitario Virgen de Las Nieves, Avenida de Las Fuerzas Armadas 2, 18014 Granada, Spain; 2 European Hidradenitis Suppurativa Foundation (EHSF), Dessau‑Roßlau, Germany; 3 Instituto de Investigación Sanitaria IBS Granada, Granada, Spain

Correction to: Scientific Reports, 10.1038/s41598-020-70176-x, published online 07 August 2020

The original version of this Article contained errors in the spelling of the authors Luis Salvador-Rodríguez, Salvador Arias-Santiago and Alejandro Molina-Leyva, which were incorrectly given as Salvador-Rodriguez Luis, Arias-Santiago Salvador & Molina-Leyva Alejandro respectively.

These errors have now been corrected in the PDF and HTML versions of the Article.

